# Genetic Variation on Chromosome 6 Influences F Cell Levels in Healthy Individuals of African Descent and HbF Levels in Sickle Cell Patients

**DOI:** 10.1371/journal.pone.0004218

**Published:** 2009-01-16

**Authors:** Lisa E. Creary, Pinar Ulug, Stephan Menzel, Colin A. McKenzie, Neil A. Hanchard, Veronica Taylor, Martin Farrall, Terrence E. Forrester, Swee Lay Thein

**Affiliations:** 1 King's College London School of Medicine, Division of Gene and Cell, Based Therapy, James Black Centre, Denmark Hill Campus, London, United Kingdom; 2 Tropical Metabolism Research Unit, Tropical Medicine Research Institute, University of the West Indies, Mona, Kingston, Jamaica; 3 National Blood Transfusion Centre, Kingston, Jamaica; 4 Department of Cardiovascular Medicine, Wellcome Trust Centre for Human, Genetics, University of Oxford, Oxford, United Kingdom; 5 King's College Hospital, Department of Haematological Medicine, Denmark Hill, London, United Kingdom; Baylor College of Medicine, United States of America

## Abstract

Fetal haemoglobin (HbF) is a major ameliorating factor in sickle cell disease. We investigated if a quantitative trait locus on chromosome 6q23 was significantly associated with HbF and F cell levels in individuals of African descent. Single nucleotide polymorphisms (SNPs) in a 24-kb intergenic region, 33-kb upstream of the *HBS1L* gene and 80-kb upstream of the *MYB* gene, were typed in 177 healthy Afro-Caribbean subjects (AC) of approximately 7% European admixture, 631 healthy Afro-Germans (AG, a group of African and German descendents located in rural Jamaica with about 20% European admixture), 87 West African and Afro-Caribbean individuals with sickle cell anaemia (HbSS), as well as 75 Northern Europeans, which served as a contrasting population. Association with a tag SNP for the locus was detected in all four groups (AC, *P* = 0.005, AG, *P* = 0.002, HbSS patients, *P* = 0.019, Europeans, *P* = 1.5×10^−7^). The association signal varied across the interval in the African-descended groups, while it is more uniform in Europeans. The 6q QTL for HbF traits is present in populations of African origin and is also acting in sickle cell anaemia patients. We have started to distinguish effects originating from European and African ancestral populations in our admixed study populations.

## Introduction

The persistence of fetal haemoglobin (HbF, α_2_γ_2_) production beyond early childhood provides a major clinical benefit in patients with sickle cell anaemia (HbSS) and β-thalassaemia. HbF is restricted to a sub-population of erythrocytes, called F cells[1], the abundance of F cells, and HbF, is subject to strict genetic control[2]. Various studies in diverse populations have established the influence of the β globin gene cluster (*HBB*) on chromosome 11p15 and the ^G^γ promoter on HbF; the causal variant here is thought to be a single-base substitution (T/C) at position −158 of the ^G^γ globin gene, termed *Xmn*I ^G^γ site[3]. Two other loci influencing F-cell levels have recently been discovered[4]–[6]; the *HMIP* locus (*HBS1L-MYB Intergenic Polymorphism*) on chromosome 6q was first discovered in an Asian-Indian family with β thalassaemia and persistence of HbF, and subsequently mapped in European samples to the interval between two genes, the *MYB* oncogene and the putative *HBS1L* [4]. The third major locus is the oncogene *BCL11A* on chromosome 2[5], [6].

In Europeans, these three genetic loci contribute nearly half of all F-cell variability: the *XmnI ^G^γ* site on chromosome 11p15 accounts for 10% of the variance; the *HMIP* locus on chromosome 6q, 19%; and *BCL11A* on chromosome 2p, 15%[5]. At the *HMIP* locus, the genetic variants reside in three linkage disequilibrium (LD) blocks, *HMIP-1*, *2* and *3*. Genetic variants in the three blocks completely account for the variance in FC levels due to the 6q QTL, but most of the effect is concentrated in *HMIP-2*. This block is characterised by eleven SNPs, which can not be resolved genetically in European subjects, because they are in strong LD with each other and show equal strength of association with the F-cell trait[4]. We decided to investigate these SNPs in African-descended populations to test whether an effect is present, and whether the haplotype make-up in African-descended populations might facilitate further resolution and eventual fine-mapping of the causative genetic variation underlying HbF and FC levels in the 6q QTL.

Here, we present results showing that an effect of the *HMIP-2* block on F cell levels can be detected in two healthy African-descended populations from Jamaica, and that the same locus also influences HbF levels in HbSS patients of African descent. Association across the 24-kb region is far more varied in African than in European haplotypes, which suggests that future fine-mapping studies will be able to pinpoint functionally active sequence variation.

## Methods

### Ethics Statement

Ethical approval was given by the University Hospital of the West Indies/University of the West Indies Faculty of Medical Sciences Ethics committee (study #21) and the Ministry of Health of Jamaica Ethics Committee (study #150); and the local King's College Hospital Ethics committee (No. 01-083).

All participants provided written informed consent

### Participants

Three population samples were recruited and phenotyped in Jamaica: 177 healthy blood donors from Kingston (Afro-Caribbean group, AC); 631 healthy subjects composed of families and unrelated individuals (Afro-German group, AG) from Seaford Town, Westmoreland; and 75 healthy European expatriates. The AC group is mostly of African descent, with an approximately 7% of European genetic ancestry[7]. The AG group is from a small population with additional European ancestry originating from 19^th^ century German immigrants, with about 20% European admixture[7], but otherwise similar to the AC group. All individuals were screened for sickle cell disease and β thalassaemia by haemoglobin electrophoresis and those who were affected were excluded from further analysis.

In a separate study, 87 HbSS patients (49 female, 38 male) of Afro-Caribbean and West African descent were recruited from the specialist clinic in the Haematology Outpatient Unit of King's College Hospital in South London. Our patient group is ethnically heterogeneous; about a third is of African-Caribbean decent and the rest of West-African origins. The extent of European admixture has not yet been measured. At the time of the study, the patients ranged from 11 to 64 years of age, with an average age of 30 years. Dates of blood transfusion were noted and no patient had been transfused during the preceding three months, or had been receiving hydroxycarbamide at the time when the HbF values were obtained.

### Phenotyping

In most healthy individuals fetal haemoglobin levels are low, with a considerable proportion of subjects having values below 0.3% HbF; in this range current HPLC measurements are very imprecise, and the traits better represented by F cells in normals. A major determinant of HbF levels is the number of HbF carrying cells, referred to as F cells which shows 89% heritability[2]. We have previously shown that there is a strong correlation between HbF and F cell levels in the range encountered in healthy individuals (Tatu, T., DPhil Thesis 2001, University of Oxford; Creary, L., PhD Thesis 2007, University of London). The proportion of F cells among erythrocytes was estimated by flow cytometry[8] using an anti γ globin antibody. For our group of HbSS patients, F-cell data are not available, but the HbF proportion of total haemoglobin is routinely measured for all patients visiting our clinic by high pressure liquid chromatography (HPLC) on a BioRad Variant II system. HbF levels encountered in HbSS patients are between 1% to 30%, values that can be measured accurately and precisely by HPLC. HbF and FC values were log transformed for the study.

### Genotyping

Genotyping for *XmnI ^G^γ* (*rs7482144*) was performed by PCR/restriction enzyme analysis[9], and for all *HMIP-2* markers, by TaqMan (Applied Biosystems, Foster City, Ca), a hybridisation based procedure. Some primers and fluorescent MGB probes were purchased from Applied Biosystems as pre-designed assays (with the assay number in brackets): for *rs9376090* (C___3119885_10), *rs11759553* (C___3119886_10), *rs9376092* (C__27440941_10), *rs9389269* (C__27440940_10), *rs9402686* (C___2737531_10), and *rs11154792* (C___3119892_10). Other Taqman assay primers and probes were custom designed: *rs4895440* (Forward: 5′–GCTGGTTATGGGAATAGAGAGTGATG–3′, Reverse: 5′–CTCACTTACTCAGTTCTCTGCTCAT–3′, CTTTACAAAGAGTCTTTCC–VIC, TTTACAAAGAGACTTTCC–FAM), *rs9399137* (Forward:5′–CATCACCTTAAAAGGCGGTATTGTATG–3′, Reverse: 5′–GATTCCACTTTCAGAACTTATCCCAAGA, AAAAACTGTGAATAACC–VIC, AAAAAACTGTAAATAACC–FAM), *rs9402685* (Forward:5′–TGAGATTACAGGCGCATGCAA–3′, Reverse:5′–ACTGAGGCAGGTGGATTGC–3′, TTCGAGAGCAACCTGA–VIC, TCGAGAGCAGCCTGA–FAM), *rs35959442* (Forward:5′–CCCAGAGCGTCCAAGGG–3′, Reverse:5′–CAAAGAACAGGTGCCTCTAGTTGT–3′, CTACAGCAGGCTTCAG–VIC, CTACAGCAGCCTTCAG–FAM), and *rs4895441* (Forward:5′–GCTGGTTATGGGAATAGAGAGTGATG–3′, Reverse:5′–GTTATCTCCCTCACTTACTCAGTTCTC–3′, CTCTTTGTAAAGTGATACATG–VIC, TCTTTGTAAAGTGGTACATG–FAM). Primers and probes were designed at the Centre National de Génotypage in Evry, France[4].

### Statistical methods

Genetic association of FC and HbF traits with *HMIP-2* marker alleles was tested by multiple regression (SPSS v.12) including covariates age, sex, and additive effects of the beta globin gene locus (i.e., the *XmnI ^G^γ* polymorphism).

Linkage disequilibrium (LD) between markers and the presentation of marker haplotypes was investigated with Haploview v.3.31[10] and Phase v2.1.1[11]. The effective number of haplotypes[12] was calculated as n_e_ = 1/Σp_i_^2^, where p_i_ are the individual haplotype frequency estimates.

## Results

**Figure 1:**
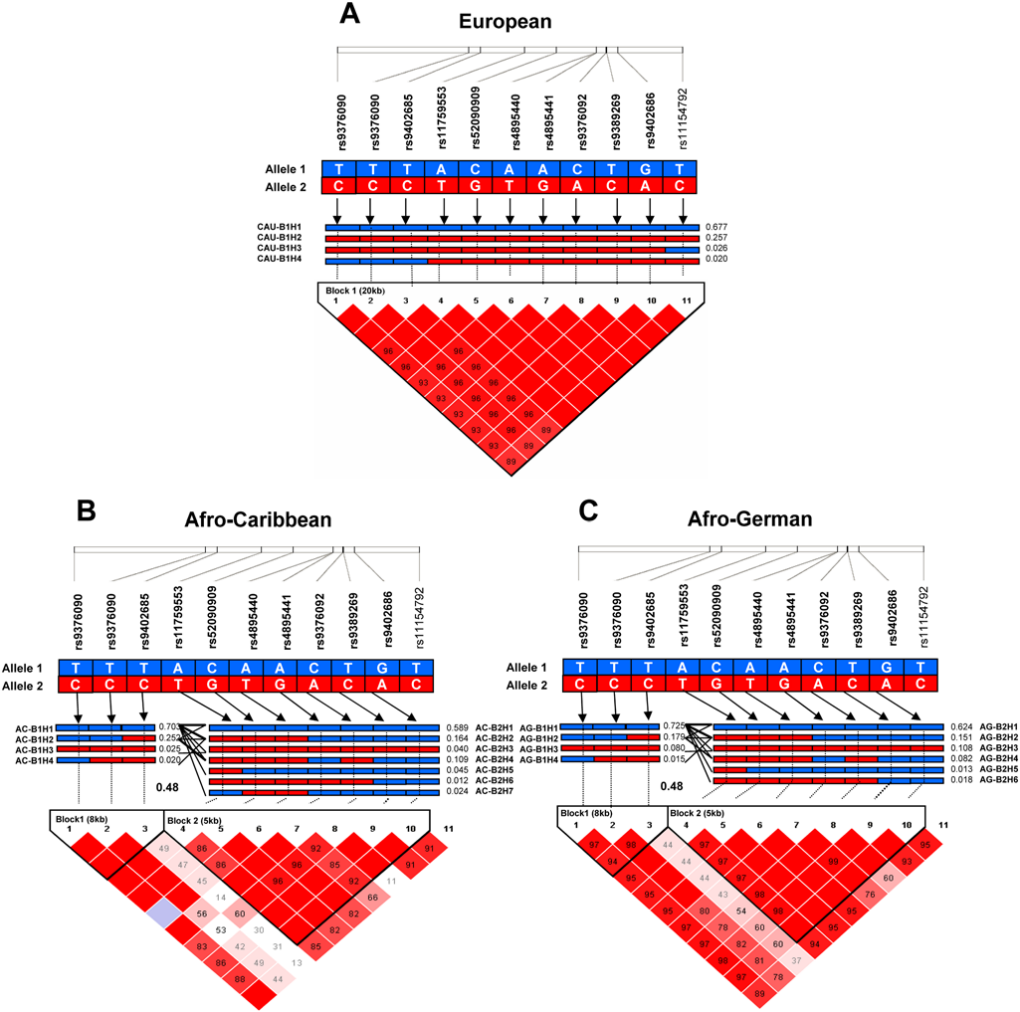
Linkage disequilibrium patterns and haplotype blocks in the Afro-Caribbean, Afro-German and Caucasian populations. Pairwise linkage disequilibrium, measured by D′, of the SNPs in the 24-kb intergenic region and common haplotype structures in the Afro-Caribbean (A), Afro-German (B), and Caucasian (C) populations. The location of each genotyped SNP located on chromosome 6q is shown on the white bar at the top of each diagram. The magnitudes of LD between respective pairs of SNPs are shown in each square. Squares without values represent complete LD (D′ = 1). The standard colour scheme of Haploview was used to display the strength of LD. Where, for LOD>2 complete LD is shown as bright red (D′ = 1), and runs through to pink (D′<1) then to white (D′ = 0). LOD<2 are represented as white squares. Haplotypes with a frequency greater than 1% are shown. Within each haplotype (H1 to H7), blue blocks represent the reference allele, whereas red blocks represent the alternative allele. Numbers next to each haplotype block are haplotype frequencies. Bold lines joining haplotypes from each block represent combined haplotypes with frequencies >0.1%, and thin lines are for frequencies <0.1%. In the crossing areas between haplotype blocks, a value of multi-allelic D′ is shown to represent the level of recombination between blocks.

Eleven SNPs which mark the *HMIP-2* block define two complementary haplotypes in European expatriates (Figure 1A); each SNP's minimum allele frequency (MAF) is approximately 30% in this population of European descent (Table 1). By contrast, MAFs in the African-descended populations ranged from 2–38% (Table 1) and there was more haplotype diversity (effective number of haplotypes n_e_ = 3.7 and 3.3 in AC and AG groups, compared to 1.9 in Europeans), with the region subdivided into two extended blocks (Figure 1B & 1C). SNP I-02 (*rs9399137*), which tags the European *HMIP-2* block[4], is relatively infrequent in African-descended populations (MAF≤9%) compared to Europeans (MAF = 29%).

**Table 1:** Healthy participants from Jamaica: Test for association of F cell levels with *HMIP-2* markers.

*HMIP-2* marker	European			Afro-Caribbean			Afro-German			Combined Jamaican Group			Combined Jamaican Group, exclusion of non-African haplotypes		
	(n = 76)			(n = 178)			(n = 633)			(n = 811)			(n = 693)		
	MAF	β	P =	MAF	β	P =	MAF	β	P =	MAF	β	P =	MAF	β	P =
I-01 (rs9376090)	0.3	0.497	**2.6×10^−7^**	0.03	0.528	**0.045**	0.08	0.161	**0.016**	0.07	0.141	**0.036**	0	n a	n a
**I-02 (rs9399137)**	0.29	0.511	**1.5×10^−7^**	0.04	0.559	**0.005**	0.09	0.196	**0.002**	0.08	0.206	**9.2×10^−4^**	0.02	0.412	**0.005**
I-03 (rs9402685)	0.29	0.512	**1.3×10^−7^**	0.30	0.051	0.579	0.27	0.039	0.341	0.28	0.051	0.183	0.23	0.005	0.908
I-04 (rs11759553)	0.31	0.559	**2.7×10^−9^**	0.38	0.135	0.122	0.37	0.123	**0.001**	0.37	0.127	**3.5×10^−4^**	0.33	0.100	**0.012**
I-05 (rs35959442‡)	0.31	0.524	**1.9×10^−8^**	0.36	0.036	0.681	0.36	0.105	**0.007**	0.36	0.089	**0.014**	0.32	0.071	0.079
I-06 (rs4895440)	0.3	0.549	**2.4×10^−9^**	0.36	0.040	0.650	0.36	0.107	**0.005**	0.36	0.091	**0.012**	0.32	0.067	0.093
I-07 (rs4895441)	0.3	0.516	**3.7×10^−8^**	0.05	0.428	**0.022**	0.12	0.181	**0.001**	0.11	0.168	**0.002**	0.05	0.144	0.099
I-08 (rs9376092)	0.3	0.516	**3.7×10^−8^**	0.18	0.169	0.161	0.21	0.102	**0.024**	0.2	0.101	**0.020**	0.15	0.056	0.292
I-09 (rs9389269)	0.3	0.549	**2.4×10^−9^**	0.04	0.540	**0.010**	0.11	0.250	**1.3×10^−5^**	0.1	0.237	**3.9×10^−5^**	0.03	0.380	**6.5×10^−4^**
I-10 (rs9402686)	0.3	0.560	**5.6×10^−9^**	0.04	0.550	**0.008**	0.1	0.242	**5.9×10^−5^**	0.09	0.234	**1.1×10^−4^**	0.03	0.403	**4.4×10^−4^**
I-11 (rs11154792)	0.3	0.484	**7.4×10^−7^**	0.13	0.097	0.440	0.15	0.225	**1.1×10^−5^** *	0.15	0.195	**6.4×10^−5^**	0.08	0.205	**0.003**

The multiple-regression model included covariates age, sex, and an additive genetic effect of the *Xmn*I ^G^γ site. The minor-allele frequency (MAF) refers to the same allele in all populations.

Genetic association was also tested in the combined Jamaican group (Afro-Caribbean and Afro-German groups combined). The last column is a repeat of this analysis, after exclusion of individuals who carry the C allele of *rs9376090* (I-01, 7 homozygotes and 95 heterozygotes), which tags non-African haplotypes at the *HMIP-2* locus. Also excluded were 16 individuals with an unknown genotype at this SNP. Hence, a total of 693 individuals were analysed in the combined Jamaican group.

* A nominally significant (*P* = 0.04) dominance effect was detected only for I-11 in the AG group and was included in the regression model for this data point.

‡ *rs35959442* was previously named *rs52090909*.

All 11 SNPs show strong association (*P*≤7×10^−7^) with FC levels in the European sample (Table 1) in a pattern consistent with a previous analysis of a European British population[4]. Multiple SNPs showed association (*P*-values range from 1.1×10^−5^–0.045) with FC and HbF levels in both healthy individuals and patients with HbSS of African descent (Table 1 and 2); unlike the European population, association was considerably variable within and between these populations. The variability in the strength of association was also evident in the magnitude of the beta-coefficients (Table 1 and 2). SNP I-02 (rs9399137) shows association with FC levels in healthy AC and AG participants (P = 0.005 and 0.002, respectively) and with HbF levels (P = 0.019) in the group of HbSS patients. Six out of eleven markers show no significant association in the AC population. In the AG population, a wide spectrum of association was observed, one marker (I-03) showed no association while markers in the distal part of the block showed strong association with P-values in the region of 10^−5^ (I-09 to I-11, Table 1). In the HbSS patients, just two markers (I-02 and I-07) showed significant association with HbF (Table 2).

**Table 2:** British patients with sickle cell anaemia (HbSS): Test for association of HbF levels with *HMIP-2* markers.

*HMIP-2* marker	All Patients			Exclusion of non-African haplotypes		
	(n = 87)			(n = 81)		
	MAF	β	P =	MAF	β	P =
I-01 (rs9376090)	0.02	0.567	0.226	n_a	n_a	n_a
**I-02 (rs9399137)**	0.07	0.642	**0.019**	0.05	0.629	**0.049**
I-03 (rs9402685)	0.31	−0.010	0.937	0.29	0.030	0.834
I-04 (rs11759553)	0.38	0.190	0.109	0.36	0.204	0.096
I-05 (rs35959442^‡^)	0.36	0.154	0.212	0.33	0.168	0.196
I-06 (rs4895440)	0.35	0.146	0.245	0.33	0.157	0.230
I-07 (rs4895441)	0.06	0.554	**0.027**	0.04	0.559	0.071
I-08 (rs9376092)	0.16	0.053	0.730	0.13	0.061	0.773
I-09 (rs9389269)	0.04	0.471	0.156	0.01	0.420	0.452
I-10 (rs9402686)	0.04	0.446	0.181	0.01	0.398	0.477
I-11 (rs11154792)	0.12	0.238	0.244	0.1	0.163	0.457

The regression is as in Table 1. Again, the analysis was repeated after exclusion of individuals who carry the C allele of *rs9376090* (I-01, 4 heterozygotes), and 2 individuals with unknown genotype at this SNP. No significant dominance effect were detected in the patients.

To examine the importance of the very strong European-tagged association in our admixed Caribbean study subjects, we repeated the above association analysis after excluding individuals who carry the C allele of the *rs9376090* (I-01) marker. According to HapMap data[13], this marker is invariant (MAF = 0) in Yoruba subjects from Nigeria and is a frequent polymorphism in populations of European (C allele frequency = f_C_ = 22%), Chinese (f_C_ = 29%) and Japanese (f_C_ = 34%) descent. Since association at the *HMIP-2* locus in European chromosomes is efficiently captured by the I-01 dimorphism[4], elimination of carriers of the C allele should eliminate the influence of non-African genetic variants at this locus. Association detected with the remaining subjects will then be derived mainly from the action of other alleles that are common in Africans, i.e. that reflect genetic variability within the African lineage or ancient alleles that are prevalent in multiple continental ancestry groups.

After elimination of individuals with the C allele at the I-02 marker, association was largely reduced, but a strong association signal persisted in the distal portion of the *HMIP-2* region (in the combined AC+AG groups: I-09, *P* = 6.5×10^−4^; I-10, *P* = 4.4×10^−04^; and I-11, *P* = 0.003, Table 1). Marginally significant variation also remained in the HbSS patients (*P* = 0.049 at I-02, Table 2).

## Discussion

We report on the association of genetic variation in a 24-kb *HBS1L*-*MYB* intergenic fragment, termed *HMIP-2*, with fetal haemoglobin traits in three admixed populations of predominant African heritage. Association with Fcell levels was detected in two healthy population samples from Jamaica and with HbF levels in a small group of patients with sickle cell anaemia from the UK. The latter result confirms a recent replication of the *HMIP-2* locus in patients with sickle cell disease from the US and Brazil[14]. Dominance effects were largely not detected (with the exception of I-11 in the AG group), which is likely due to a lack of statistical power and of homozygotes for the minor allele in African-descended populations.

A strong effect of this genomic region was originally seen in Europeans[4], [6]. The replication of these results in individuals of African descent is significant for two reasons. First, a large proportion of people with sickle cell disease, a disease which benefits from raised HbF, have predominantly African ancestry. Second, the study of multiple populations, especially those from Africa, offers insights into genetic mechanisms and might help reduce the size of the region of causative variation. Investigating healthy individuals, in addition to patients with HbSS, removes some of the confounding factors and genetic complexity that contribute to the HbF phenotype in sickle cell disease patients.

In our efforts towards fine-mapping the *HMIP-2* region, the analysis of African-descended populations has provided suggestive but not conclusive answers. Our data suggest that a distinct association signal originating from African chromosomes is marked by SNPs in the distal proportion of *HMIP-2* (I-09 to I-11). While strength of association for indirect markers is not indicative of the specific location of the true functional variants, our eleven SNPs should also be evaluated as potentially functional variants themselves. In this respect it appears that I-03, which has virtually no effect in any of the African-descended populations, is unlikely to be a causative variant. Genetic admixture tends to confound fine-mapping through the introduction of extended LD and additional allelic heterogeneity. We have addressed this problem by excluding individuals with non-African active haplotypes at the *HMIP-2* locus; ultimately the investigation of a much larger sample size of individuals with exclusively African ancestry may be a better approach.

On the other hand, admixed populations offer specific mapping opportunities for genetically complex diseases that vary in prevalence across the ancestral populations[15], [16]. It can also allow the observation of known functional genetic variation in a different population background. Alleles may differ between similar populations, not only in frequency, but also in effect size: the relatively small effect of I-01-tagged European haplotypes in AG (β = 0.16, compared to 0.5 in Europeans and 0.53 in the AC group) is unexplained and might be due to a specific founder effect connected with the history of this community. Another interesting feature of the I-01 marker is that its allele C, which increases the number of F cells, has a lower frequency in the AC population when compared to the Europeans, yet the AC population has higher F cell levels than in the European population. In conclusion, genetic effects on F cells in admixed populations might differ from their assumed parental populations in ways that cannot be predicted easily.

We have also included in this report our first evaluation of HbF levels in a sickle cell patient population. While HbF and F cells are closely related traits, the genes that regulate each might differ, especially in the strength of their relative effects. Encouraging in this context is the fact that genome-wide association studies of F cell levels in non-anaemic individuals[5] and of HbF[6] have identified the same set of three major loci, including *HMIP-2*. We and others have now shown that the F cell locus *HMIP-2* also influences HbF levels in patients with HbSS[14]. The statistical power provided by our small and heterogeneous group of HbSS patients does not allow much more than simple detection of the effect of the overall locus. The drop in association seen after exclusion of non-African effects seems to indicate that European alleles contribute to the variance of FC levels in admixed patients, but the independent association with African-derived polymorphism remains.

With this study we have shown that the *HMIP-2* locus appears to influence HbF-related traits in healthy individuals and in patients with sickle cell anaemia of African origin. Our initial results suggest that further extended studies of these populations complemented by studies of participants from the African continent itself may be a powerful approach for identifying loci involved in the determination of HbF persistence and other quantitative traits.
